# Indicaxanthin from *Opuntia ficus indica* (L. Mill) Inhibits Oxidized LDL-Mediated Human Endothelial Cell Dysfunction through Inhibition of NF-*κ*B Activation

**DOI:** 10.1155/2019/3457846

**Published:** 2019-02-18

**Authors:** Alessandro Attanzio, Anna Frazzitta, Rosalia Busa', Luisa Tesoriere, Maria A. Livrea, Mario Allegra

**Affiliations:** Dipartimento di Scienze e Tecnologie Biologiche, Chimiche e Farmaceutiche (STEBICEF), Università degli Studi di Palermo, Via Archirafi 28, 90123 Palermo, Italy

## Abstract

Oxidized low-density lipoproteins (oxLDL) play a pivotal role in the etiopathogenesis of atherosclerosis through the activation of inflammatory signaling events eventually leading to endothelial dysfunction and senescence. In the present work, we investigated the effects of indicaxanthin, a bioavailable, redox-modulating phytochemical from *Opuntia ficus indica* fruits, with anti-inflammatory activity, against oxLDL-induced endothelial dysfunction. Human umbilical vein cord cells (HUVEC) were stimulated with human oxLDL, and the effects of indicaxanthin were evaluated in a range between 5 and 20 *μ*M, consistent with its plasma level after a fruit meal (7 *μ*M). Pretreatment with indicaxanthin significantly and concentration-dependently inhibited oxLDL-induced cytotoxicity; ICAM-1, VCAM-1, and ELAM-1 increase; and ABC-A1 decrease of both protein and mRNA levels. From a mechanistic perspective, we also provided evidence that the protective effects of indicaxanthin were redox-dependent and related to the pigment efficacy to inhibit NF-*κ*B transcriptional activity. In conclusion, here we demonstrate indicaxanthin as a novel, dietary phytochemical, able to exert significant protective vascular effects *in vitro*, at nutritionally relevant concentrations.

## 1. Introduction

Atherosclerosis is a long, multifactorial, inflammatory process characterized by the accumulation of lipids in the wall of large- and medium-sized arteries [1, 2]. According to the oxidative theory of atherosclerosis, and its modifications over the last decade, hypercholesterolemia is one of the major risk factors of this condition as it triggers the accumulation of oxidized low-density lipoproteins (oxLDL) in the subintimal space [1, 2]. In areas at risk of atherosclerosis (e.g., at arterial bifurcations), the turbulent blood flow induces an endothelial activation, characterized by a local inflammatory response that generates an endocellular oxidative and nitrosative stress [3]. The increased levels of reactive oxygen and nitrogen species (RONS) activates selected redox-dependent transcription factors, such as NF-*κ*B, that in turn induce an overexpression of endothelial adhesion molecules such as ICAM-1, VCAM-1, and ELAM-1 [1, 2]. This phenomenon increases the adhesion of leukocytes that eventually transmigrate in the subendothelial space where they are converted in macrophages. At the same time, the enhanced endothelial permeability favors the influx of LDL into the subendothelial intima where the inflammatory cells generate RONS inducing a progressive LDL oxidation. oxLDL, then, interact with endothelial cells (EC), disrupt the antioxidant defences of vascular endothelium, and boost leukocyte chemotaxis, reinforcing the whole inflammatory reaction that leads to a persistent EC dysfunction. As a consequence, a chronic systemic inflammatory response is established and eventually results in the proliferation of macrophagic foam cells and the formation of fatty streaks, the hallmark of the early atherosclerotic lesions [3].

In line with the key role of LDL in atherosclerosis, ATP-binding cassette A1 (ABC-A1), the cholesterol efflux system present in all tissues, endothelium included, has been demonstrated to offer antiatherogenic protection [4, 5]. Indeed, under physiological conditions, synthesized cholesterol can efflux from EC through ABC-A1 to maintain cholesterol homeostasis. Disturbed flow and oxLDL inhibit ABC-A1-mediated cholesterol efflux. As a result, cholesterol is removed through other pathways which may lead to lipid deposition in the subendothelial space [4–6].

Notwithstanding the solid basis of the oxidative theory of atherosclerosis, the vast majority of clinical trials and all meta-analyses surprisingly conclude that antioxidant vitamin supplementation has no beneficial effect on cardiovascular events. On the other hand, the association between “Mediterranean diet” and reduction of major chronic degenerative diseases, including the cardiovascular ones, appears robust and conclusive [1, 7, 8], suggesting the involvement of other redox-dependent molecular controls. In comparison to other fruits within the “Mediterranean diet” (e.g., oranges, lemons, and grapes), cactus pear (*Opuntia ficus indica*, L Mill) fruit is one that is less consumed and less studied, notwithstanding its abundance throughout selected areas of Italy, Spain, Greece, and North African countries.

**Figure 1:**
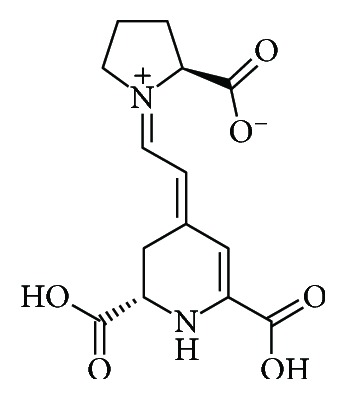
Chemical structure of indicaxanthin.

Indicaxanthin (Figure 1), a betalain pigment from cactus pear fruit, has been the object of sound experimental work over the last years [9]. As with many phytochemicals, it is a redox-active compound and has been shown to act as antioxidant in a number of *in vitro* studies [9]. Interestingly, thanks to its charged portions, ionizable groups, and lipophilic moieties, it is amphiphilic [10] and has been demonstrated to interact with cell membranes [11]. This feature is critical to allow bioactive compounds to interact with cells and initiate signaling events. In this regard, indicaxanthin has been shown to modulate specific redox-dependent signaling pathways involved in macrophage activation and apoptosis and epithelial and endothelial dysfunction *in vitro* [9, 12, 13]. Remarkably, and in contrast with the majority of dietary phytochemicals, indicaxanthin is highly bioavailable [14]. The molecule has been shown to cross the unaltered intestinal epithelial cell *in vitro* being absorbed through paracellular junctions [11]. In line with that, indicaxanthin has been found in human plasma at a 7 *μ*M peak concentration 3 h after the ingestion of four cactus pear fruits containing 28 mg of the pigment [14]. Moreover, its amphiphilicity allows it to cross the blood-brain barrier, located within the CNS, and modulate its bioelectric activity [15]. Finally, thanks to its bioavailability and redox-modulating properties, indicaxanthin exerts significant pharmacological effects *in vivo*. Indeed, oral administration of the phytochemical at nutritionally relevant doses (2 *μ*mol/kg) generates, in rats, a plasma peak concentration of 0.2 *μ*M able to exert strong anti-inflammatory effects in a model of acute inflammation [16].

In the light of the strong interconnections between inflammation, atherosclerosis, and diet, and the anti-inflammatory and redox-modulating properties of indicaxanthin, the aim of this work was to evaluate the effects of the phytochemical, at nutritionally relevant concentrations, in an *in vitro* model of innate immunity activation represented by oxLDL-induced endothelial dysfunction. To this end, we investigated whether indicaxanthin prevented the oxLDL-mediated adhesion molecule overexpression and ABC-A1 downregulation, in human cultured EC. Mechanistic details of the redox-dependent NF-*κ*B transcriptional activity have also been investigated.

## 2. Materials and Methods

### 2.1. Reagents

All reagents, unless otherwise stated, were from Sigma-Aldrich (Milan, Italy) and of the highest available purity grade.

### 2.2. Extraction and Purification of Indicaxanthin from Cactus Pear Fruits

Indicaxanthin was isolated from cactus pear (*Opuntia ficus indica*) fruits (yellow cultivar) as previously described [16].

### 2.3. Isolation and Oxidation of Human LDL

Blood samples from healthy subjects with informed consent were obtained after an overnight fasting. LDL were isolated from plasma as previously described [17] and diluted to 100 *μ*g protein/ml in PBS. Protein concentration of the particle was evaluated by Bradford assay as reported elsewhere [17].

Aliquots of native LDL (nLDL) were then subjected to lipid oxidation by treatment with 5 *μ*M CuSO_4_ at 37°C for 24 h, as previously described [14].

The oxidation state of LDL, in terms of conjugated dienes (CD) hydroperoxides and thiobarbituric acid-reactive substances (TBARS), was evaluated as previously reported [14].

oxLDL were then concentrated with centrifugal filter devices according to the manufacturer's instructions (Millipore, Milan, Italy) to a value of 100 *μ*g protein/ml. All samples (nLDL and oxLDL) were filter-sterilized (0.2 *μ*m Millipore filters, Milan, Italy), aliquoted, and stored at -80°C up to one month.

### 2.4. Cell Culture

HUVEC (human umbilical vein endothelial cells) were purchased from Lonza (Milan, Italy), grown in endothelial growth medium (Lonza) at 37°C in a humidified incubator under 5% CO_2_, subcultured by trypsinization, and used up to passage 4.

### 2.5. HUVEC Treatment

In order to perform the experiments, cells were seeded in 6-well plates at a density of 2 × 10^4^ cells/well. Upon 80% confluence, HUVEC were incubated, after overnight starving, either in the absence (control cells) or in the presence of 100 *μ*g oxLDL/ml for 16 h in a serum-free endothelial basal medium (Lonza, Milan, Italy). When necessary, HUVEC were pretreated for 1 h with indicaxanthin and then stimulated with oxLDL as described above.

### 2.6. Cell Viability Test

Cytotoxicity of oxLDL was assessed through MTT [3-(4,5-dimethylthiazol-2-yl)-2,5-diphenyl-2*H*-tetrazolium bromide] conversion assay according to the manufacturer's instructions (Invitrogen, Milan, Italy). Cell viability was expressed as percentage of the absorbance value measured in control HUVEC.

### 2.7. Flow Cytometry Analysis

At the end of the incubation time, HUVEC were washed with PBS, harvested with cell dissociation medium, and diluted in a washing buffer containing PBS, 0.1% bovine serum albumin, and 1 mM CaCl_2_. Aliquots (0.1 to 0.5 × 10^6^ cells in 20 *μ*l) were seeded in a 96-well plate and incubated at 4°C with 2 *μ*g/well of a mouse anti-human ICAM-1 or VCAM-1 or ELAM-1 or ABC-A1 monoclonal antibody FITC-conjugated (Invitrogen, Milan, Italy). After 1 h at 4°C, cells were washed and analysed for fluorescence on a flow cytometer (Epics II, Beckman Coulter, US). At least 20000 events were analysed for each sample. Data are expressed as median fluorescence intensity (MFI) units, measured in the FL1 (green) channel with a 488 nm (excitation) and 530 nm (emission) filter set.

RONS concentration was evaluated by staining cells with 2′,7′–dichlorofluorescin diacetate dye (DCFDA) as reported elsewhere [18].

### 2.8. Quantitative Real-Time Reverse-Transcription Polymerase Chain Reaction

Total RNA was isolated by employing an RNeasy mini kit in accordance with the manufacturer's instructions (QIAGEN, Milan, Italy). RNA (5–10 *μ*g) was then reverse-transcribed to cDNA using Superscript III Reverse Transcriptase following the manufacturer's protocol (Invitrogen, Milan, Italy) and stored at -20°C until tested. Real-time PCR for ICAM-1, VCAM-1, ELAM-1, or ABC-A1 and glucose-6-phosphate dehydrogenase (G6PDH) was carried out with an RT Real-Time™ SYBR Green PCR Master Mix and the RT^2^ PCR Primer Set in accordance with the manufacturer's instructions (SuperArray, Milan, Italy) using an ABI PRISM 7700 Sequence Detection System (Applied Biosystems, Warrington, UK). The following primers were used in the present study: human VCAM-1: (forward) 5′-CTTAAAATGCCTGGGAAGATGGT-3′, (reverse) 5′-GTCAATGAGACGGAGTCACCAAT-3′; human ICAM-1: (forward) 5′-GGCTGGAGCTGTTTGAGAAC-3′, (reverse) 5′-CTGACAAGTTGTGGGGGAGT-3′; human ELAM-1: (forward) 5′-GCCTGCAATGTGGTTGAGTG-3′, (reverse) 5′-ACGAACCCATTGGCTGGATT-3′; human ABC-A1: (forward) 5′-TGTCCAGTCCAGTAATGGTTCTGT-3′, (reverse) 5′-CGAGATATGGTCCGGATTGC-3′); and human GAPDH: (forward) 5′-CCACATCGCTCAGACACCAT-3′, (reverse) 5′-CCAGGCGCCCAATACG-3. Thermal cycling conditions included a prerun of 2 min at 50°C and 15 min at 95°C followed by 40 cycles at 95°C for 30s, 55°C for 30s, and 72°C for 30s. RT^2^-PCR data were quantified as cycle of threshold (Ct) values, and the relative expression of each gene was normalized to housekeeping gene GAPDH.

### 2.9. Reporter Gene Assay

NF-*κ*B activity was examined by transfecting HUVEC with pNF*κ*B-Luc luciferase construct (Stratagene, CA, USA) as reported elsewhere [19]. Transfected HUVEC were then cocultured with 100 *μ*M oxLDL in the absence or in the presence of indicaxanthin as detailed above. After 6 h, cells were washed with PBS and lysed for 5 min at 4°C using a lysis buffer according to the manufacturer's instructions (Promega, WI, USA). Luciferase activity is expressed as relative luminescence units (RLU) × 10^3^ following reading in a TD/2020 luminometer (Turner Biosystems, CA, USA).

### 2.10. Statistical Analysis

Data from all experiments are reported as mean ± SD. Data were analysed and presented using GraphPad Prism software (GraphPad). Comparisons between two values were made using Student's *t*-test, and *P* < 0.001 were designated with triple asterisks. Multiple comparisons were performed by one-way analysis of variance (ANOVA) followed by Tukey's correction. Significance was accepted when the null hypothesis was rejected at the *P* < 0.001 level.

## 3. Results

### 3.1. Oxidative State Assessment of oxLDL

**Figure 2:**
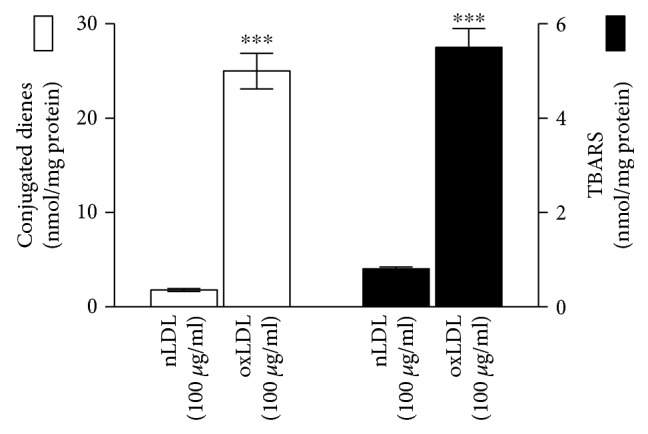
Amount of CD hydroperoxides and TBARS in the oxLDL employed in the study in comparison with untreated ones (nLDL). Values are the mean ± SD of three separate experiments carried out in duplicate; ^∗∗∗^*P* < 0.001.

The oxidative state of oxLDL was evaluated by assaying CD hydroperoxides and TBARS levels. When compared to nLDL, treatment with 5 *μ*M CuSO_4_ for 24 h determined a significant increase (*P* < 0.001) of both CD and TBARS in the particle (Figure 2).

### 3.2. Effects of Indicaxanthin on the Viability of oxLDL-Treated EC

**Figure 3:**
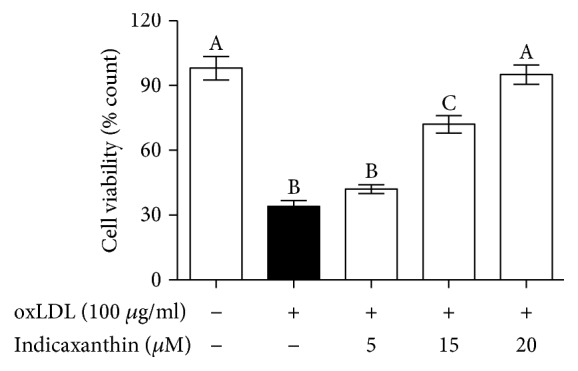
Protective effects of indicaxanthin against oxLDL-induced cytotoxicity in EC. Values are the mean ± SD of three separate experiments carried out in triplicate. Labeled means without a common letter differ with *P* < 0.001.

Incubation of HUVEC with 100 *μ*g oxLDL/ml for 16 h determined a significant decrease (*P* < 0.001) of cell viability as compared to control EC (Figure 3). On the other hand, pretreatment of HUVEC with indicaxanthin at 5 *μ*M did not improve cell viability, while at 15 *μ*M it caused a significant (*P* < 0.001) inhibition of cell death that was completely prevented with 20 *μ*M indicaxanthin (Figure 3).

### 3.3. Effects of Indicaxanthin on oxLDL-Induced Protein and mRNA Upregulation of Adhesion Molecules in EC

**Figure 4:**
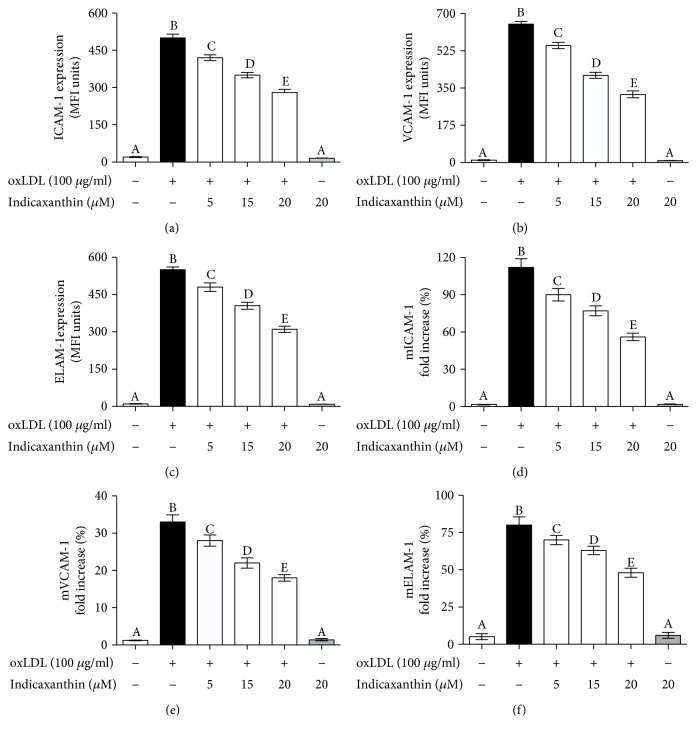
ICAM-1, VCAM-1, and ELAM-1 protein (a–c) and mRNA (d–f) levels of HUVEC stimulated with oxLDL, either in the absence or in the presence of indicaxanthin at different concentrations for 16 h. Values are the mean ± SEM of three separate experiments carried out in triplicate. Labeled means without a common letter differ; *P* < 0.001.

Incubation of HUVEC with 100 *μ*g oxLDL/ml for 16 h induced a significant (*P* < 0.001) increase of ICAM-1, VCAM-1, and ELAM-1 regarding both protein and mRNA levels (Figures 4(a)–4(f), respectively) in comparison with control cells. Noteworthy, pretreatment of EC with indicaxanthin in a concentration range between 5 and 20 *μ*M caused a significant (*P* < 0.001) concentration-dependent decrease of all the above-cited adhesion molecules regarding both protein and mRNA levels (Figures 4(a)–4(f), respectively). Importantly, incubation of EC with 20 *μ*M indicaxanthin in the absence of oxLDL did not modify the adhesion molecule, regarding both protein and mRNA levels, in comparison with control HUVEC.

### 3.4. Effects of Indicaxanthin on oxLDL-Induced Downregulation of ABC-A1 in EC

**Figure 5:**
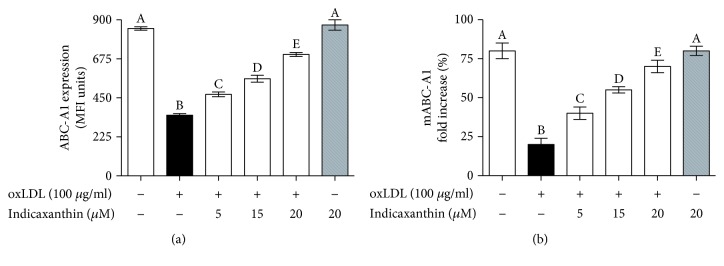
ABC-A1 protein (a) and mRNA (b) levels in HUVEC stimulated with oxLDL, either in the absence or in the presence of indicaxanthin at different concentrations for 16 h. Values are the mean ± SEM of three separate experiments carried out in triplicate. Labeled means without a common letter differ; *P* < 0.001.

Stimulation of EC with 100 *μ*g oxLDL/ml induced a significant (*P* < 0.001) decrease of ABC-A1 regarding both protein and mRNA levels, in comparison with control HUVEC (Figures 5(a) and 5(b), respectively). Noteworthy, pretreatment of EC with indicaxanthin in a range between 5 and 20 *μ*M caused a significant (*P* < 0.001) concentration-dependent increase of ABC-A1 expression, regarding both protein and mRNA levels (Figure 5). On the other hand, incubation of EC with 20 *μ*M indicaxanthin in the absence of oxLDL did not modify ABC-A1 at both protein and mRNA levels, in comparison with control HUVEC.

### 3.5. Effects of Indicaxanthin on oxLDL-Induced RONS Production

**Figure 6:**
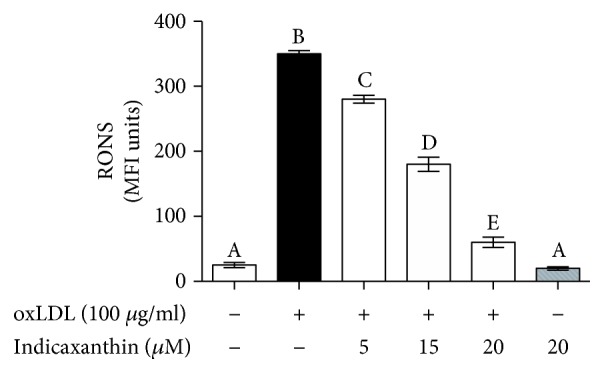
RONS levels in HUVEC stimulated with oxLDL, either in the absence or in the presence of indicaxanthin at different concentrations for 16 h. Values are the mean ± SEM of three separate experiments carried out in triplicate. Labeled means without a common letter differ; *P* < 0.001.

In comparison with control HUVEC, stimulation of EC with 100 *μ*g oxLDL/ml induced a significant increase of RONS levels (*P* < 0.001) (Figure 6). On the other hand, pretreatment of EC with indicaxanthin in a concentration range between 5 and 20 *μ*M caused a significant (*P* < 0.001) concentration-dependent decrease of endocellular RONS production when compared to control EC (Figure 6). Conversely, incubation of EC with 20 *μ*M indicaxanthin in the absence of oxLDL did not modify RONS levels, in comparison with control HUVEC.

### 3.6. Effects of Indicaxanthin on oxLDL-Induced NF-*κ*B Transcriptional Activity

**Figure 7:**
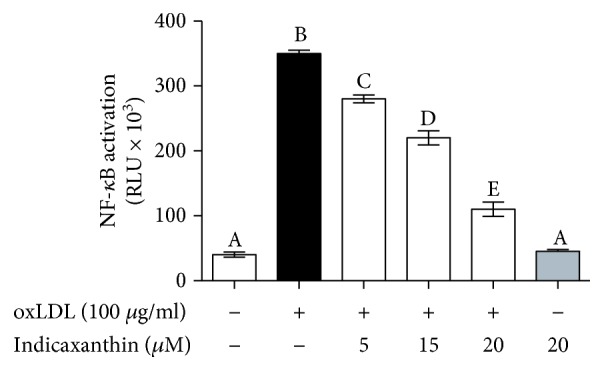
NF-*κ*B transcriptional activity in HUVEC stimulated with oxLDL, either in the absence or in the presence of indicaxanthin at different concentrations for 16 h. Values are the mean ± SEM of three separate experiments carried out in triplicate. Labeled means without a common letter differ; *P* < 0.001.

Stimulation of EC with 100 *μ*g oxLDL/ml induced a significant (*P* < 0.001) increase of NF-*κ*B transcriptional activity, in comparison with control HUVEC (Figure 7). Noteworthy, pretreatment of EC with indicaxanthin in a concentration range between 5 and 20 *μ*M caused a significant (*P* < 0.001) concentration-dependent decrease of the transcriptional activity (Figure 7). On the other hand, incubation of EC with 20 *μ*M indicaxanthin in the absence of oxLDL did not modify the transcriptional activity of NF-*κ*B, in comparison with control HUVEC.

## 4. Discussion

The present investigation falls within the intense research on the interplay between diet and immune function. The identity, the number, and the mechanisms of action of the protective agents we daily assume through our diet remain largely unknown and require further research. However, before defining appropriate clinical trials, it is crucial to evaluate, through suitable *in vitro* assays, the health-promoting effects of plant-derived compounds in order to identify the most promising ones [1–3].

In line with this perspective, we investigated here the protective effects exerted by indicaxanthin from cactus pear fruits, at nutritionally relevant concentrations, in an *in vitro* model of innate immunity activation represented by oxLDL-induced endothelial dysfunction. Our findings demonstrate that indicaxanthin protects EC from oxLDL-induced damage *in vitro*, as evaluated by an increase of the viability of EC pretreated with the phytochemical in the range between 15 and 20 *μ*M. ICAM-1, VCAM-1, and ELAM-1 overexpression is a key event in the onset and the development of EC dysfunction. Relevantly, it not only increased indicaxanthin cell viability but also preserved cellular functions, counteracting the oxLDL-induced adhesion molecule overexpression, from 5 to 20 *μ*M. From a mechanistic perspective, these effects were mediated by a modulation of gene expression that led to a downregulation of ICAM-1, VCAM-1, and ELAM-1 mRNA levels.

As stated above, many phytochemicals have shown immunomodulatory effects, *in vitro*. However, a major limitation for the employment of these compounds is their low bioavailability. On the contrary, present results appear of interest as indicaxanthin is effective at nutritionally relevant concentrations, achievable in humans after the ingestion of the fruit [14].

It is well established that endothelial adhesion molecule expression is under control of several redox-regulated transcription factors, including the master minder NF-*κ*B [20]. Moreover, oxLDL have been demonstrated to activate various transcription factors, NF-*κ*B included. In line with this, we demonstrated here an inhibition by indicaxanthin of the transcriptional activity of NF-*κ*B. This result appears consistent with our previously reported findings on the NF-*κ*B-dependent immune-modulatory and anti-inflammatory effects of indicaxanthin both *in vivo* and *in vitro*. Relevantly, the inhibition of the NF-*κ*B transcriptional activity appears to be strictly consistent with the ability of indicaxanthin to inhibit oxLDL-induced endocellular oxidative and nitrosative stress. Along these lines, we therefore propone that the phytochemical counteracted the oxLDL-induced adhesion molecule overexpression by inhibiting NF-*κ*B transcriptional activity, through a redox-dependent mechanism.

Along with ICAM-1, VCAM-1, and ELAM-1 overexpression, disruption of the reverse cholesterol transfer across the endothelium, via ABC-A1 downregulation, represents another key aspect of EC dysfunction and cholesterol deposition within the arterial wall. It is well known that oxLDL decreases ABC-A1 levels in EC via inhibiting liver receptor X (LXR) [4]. We found here that indicaxanthin counteracted the oxLDL-induced ABC-A1 downregulation. The observed inhibitory effects of indicaxanthin of the transcriptional activity of NF-*κ*B can also be connected with its ability to counteract the oxLDL-induced ABC-A1 downregulation. Indeed, several lines of evidence show that ABC-A1 can be upregulated, rather than through the activation of the LXR pathway, *via* the inhibition of the redox-sensitive ERK/NF-*κ*B pathway [21–25].

Finally, notwithstanding that indicaxanthin at 5 *μ*M does not improve EC viability, it does, however, significantly reduce adhesion molecule overexpression, NF-*κ*B transcriptional activity, and RONS production and increases ABCA-1 expression.

## 5. Conclusions

Evaluating the activities of healthy, plant-derived compounds to select the most promising ones through suitable *in vitro* assays is imperative before defining appropriate clinical trials. We demonstrated here that indicaxanthin, a bioavailable, redox-modulating phytochemical with anti-inflammatory activity, is able to exert remarkable protective effects in an *in vitro* model of vascular inflammation, at nutritionally relevant concentrations. In light of its ability to modulate specific endothelial genes involved in leukocyte adhesion and cholesterol transport, through a redox-dependent, NF-*κ*B-mediated mechanism, indicaxanthin might be further investigated *in vivo* as a potential endothelial-protective agent of dietary origin.

## Data Availability

The data used to support the findings of this study are available from the corresponding author upon request.
